# Potentially toxic elements in different tissues of great cormorant (*Phalacrocorax carbo*) at a wetland area

**DOI:** 10.1007/s11356-023-30791-3

**Published:** 2023-11-09

**Authors:** József Lehel, Adrienn Grúz, András Bartha, László Menyhárt, Rita Szabó, Kocsner Tibor, Péter Budai

**Affiliations:** 1 Department of Food Hygiene, University of Veterinary Medicine Budapest, István u. 2, Budapest, H-1400 Hungary; 2 National Laboratory for Infectious Animal Diseases, Antimicrobial Resistance, Veterinary Public Health and Food Chain Safety, University of Veterinary Medicine Budapest, István u. 2, Budapest, H-1400 Hungary; 3 Medpace Hungary Kft., Csörsz u. 49-51, Budapest, H-1124 Hungary; 4 Department of Animal Hygiene, Herd Health and Mobile Clinic, University of Veterinary Medicine Budapest, István u. 2, Budapest, H-1400 Hungary; 5 Institute of Mathematics and Basic Science, Georgikon Campus, Hungarian University of Agriculture and Life Sciences, Deák F. u. 16, Keszthely, H-8360 Hungary; 6 Department of Plant Protection, Institute of Plant Protection, Georgikon Campus, Hungarian University of Agriculture and Life Sciences, Deák F. u. 16, Keszthely, H-8360 Hungary; 7 Digital Food Chain Education, Research, Development and Innovation Institute, University of Veterinary Medicine Budapest, István u. 2, Budapest, H-1400 Hungary

**Keywords:** Great cormorant, Bone, Feather, Kidney, Liver, Potentially toxic elements

## Abstract

Concentrations of 4 potentially toxic elements (As, Cd, Hg, Pb) were investigated in the feather, liver, kidney, and bone of great cormorants (*Phalacrocorax carbo*). The tissue samples were taken at the Central Tisza - Jászság Nature Conservation Area in Hungary. They were analysed by inductively coupled plasma optical emission spectroscopy (ICP-OES). The goal of the investigation was to analyse the metal burden of the above-mentioned elements in the various tissues of these wild birds and to provide important information for monitoring the environmental pollution.

Amongst the examined potentially toxic elements no statistical gender difference was observed, so the data were not separated based on them during the statistical analysis. The concentration of mercury was the highest in the feather, followed by the liver, kidney, and bone. The lead was detected in the feather with the highest level followed by the kidney, liver, and bone. The cadmium was determined in all investigated tissues with the next descending order: kidney > bone > liver > feather. Highest arsenic concentration was measured in the feather, followed by liver, kidney, and bone with the same concentration.

The detected concentrations of the investigated potentially toxic elements in different tissues of great cormorants (feathers, liver, kidney, bone) means that the living area of this birds is not highly contaminated to induce health problems or toxic signs, or even other undesirable effect in the animals.

**Supplementary Information:**

The online version contains supplementary material available at 10.1007/s11356-023-30791-3.

## Introduction

Potentially toxic metals can be found naturally in all ecosystems and released to the environment from many different anthropogenic sources such as industrial and agricultural activities (e.g. waste materials, mining processing, incineration, traffic) in various concentration (Hazrat et al. 2019). These elements that accumulate in the food net cause possible adverse effects on environmental and human health; thus, they have very important impact on the protection of the environment. These adverse effects on the environmental and health depend mostly on the mobility of metals through the different compartments (e.g. sediment, water, air) of the environment and the pathways to get into the body of a human or animals. A lot of research has been carried out to report and assess the behaviour of these metals in the environment (AMAP 1998; Hazrat et al. 2019). Based on the outcomes of these studies, the application of a lot of metals as plant protection products has been banned by the European and other commissions.

The bioaccumulation of each metal in the body of an animal depends on various factors such as biotic ones (body dimensions and mass, age, sex, diet, metabolism, and its position in the food chain) and abiotic ones (distribution of metals in its environment, salinity, temperature, and water pH, habitat type, and metal-metal interactions). But amongst all these factors, diet has the biggest influence. Usually, larger animals that are at the end of a food chain have higher metal concentrations in their tissues than smaller organisms they feed on (Catsiki et al. 1994; Al-Yousuf et al. 2000; Canli and Atli 2003; Storelli et al. 2005). So e.g. the consumption of fish exposed to different toxic heavy metals raises concerns and risks for health, not only for humans, especially in more sensitive groups of the human population (women, children), but also for birds that consume these fish (Hazrat et al. 2019).

Through contaminated water and food birds are directly exposed to potentially toxic metals and other contamination, because metals mostly enter the animals via their gastrointestinal system, and respiratory system and their skin (D’Haese et al. 2017). For most metals, the transport proteins of different biological membranes that these must pass through to get into the body have been identified already. These proteins are like transporters or molecules in the channel of cell membranes based on the selective binding receptors for the transportation of only one type of metal, not for all. These elements can be delivered to the organs and tissues via the bloodstream. From the digestive tract, the metals can be first transported to the liver by the portal circulation and then to the systemic circulation from there. Generally, the metals in the circulation can be bound to the erythrocytes or different plasma constituents (e.g. lead and organomercurials are delivered by erythrocytes, whilst inorganic mercuric derivatives and cadmium are bound to albumin section) (Lehel and Laczay 2011).

Different tissues (e.g. muscles, bones), organs (kidneys, liver), and the egg or feather of the birds can be used to analyse the difference at the level of distribution and accumulation of the chemical agents in the body of the birds (Burger and Gochfeld 2000a; Markowski et al. 2013; Kim et al. 2019; Mukhtar et al. 2020).

Deposition and release of metals take place in bones slowly during the mineral metabolism of homoiotherm vertebrates (Sánchez et al. 1987; Brandão-Neto et al. 1995; King et al. 2000), which is why the use for biomonitoring and ecotoxicological studies is not so frequent, compared to the kidneys and liver. Since these organs play a key role in the detoxification processes of metals, e.g. cadmium (Cd), lead (Pb), and mercury (Hg), and can accumulate in the higher concentrations of various metals in a relatively short time (Wapnir 1998; Myklebust and Pedersen 1999; King et al. 2000; Barjaktarovic et al. 2002; Stout et al. 2002), these tissues are studied more commonly.

It is important to know that Cd mostly accumulates in higher concentration primarily in the kidneys (Chen et al. 2021) and only in a relatively insignificant concentration in muscle. Hg is accumulated mostly in the liver (Teunen et al. 2022) and Pb in bone (Rădulescu and Lundgren 2019).

Hard tissues (e.g. bones, feathers) can provide useful information about the accumulation and multiannual exposure to different metals. Bones are found to be a good example, due to the high affinity of Pb to bones (Pain et al. 2005; Swaileh and Sansur 2006; Ethier et al. 2007), the accumulation over a lifetime, and the effect on the nervous system (Pain 1996; Kalisinska 2000).

Roberts (1981) reported that liver in birds has a great potential to indicate the change of exposure via uptake from food because increases in the concentration measured in liver show potential adverse effects on the health of the birds.

Feathers is a useful indicator of different pollutants, since during the feather’s formation the concentration of elements can be agreed with the quantities found in them. Nowadays the use of feather tissue samples for biomonitoring is becoming a more common method since the bird biology is well known, they have quite a long life span, and they can be found and feed at various levels of the food chain (Abbasi et al. 2015; Burger and Gochfeld 2016; Grúz et al. 2015, 2018, 2019; Hamza et al. 2021).

The accumulated metals in the organs of birds, especially at high levels, can be harmful to their reproduction, survival, breeding, growth, moulting, and migration (Hutton 1981; Honda et al. 1985; Savinov et al. 2003; Canova et al. 2020). Because of these reasons, birds are the useful indicators of local, regional, and global metal contamination (Burger and Gochfeld 2016). Besides, the comparison of local species and the ones that migrate can provide us important information (Frederick et al. 1999).

The objective of this study was to investigate the metal burden of the liver, kidneys, bones, and feather tissues of great cormorants (*Phalacrocorax carbo*) and thus the possible metal pollution in their feeding and nesting area at the Central Tisza - Jászság Nature Conservation Area of the Hortobágy National Park Directorate. Furthermore, the aim was to be able to obtain information about the possible contamination of the most important potentially toxic elements, i.e. heavy metals, in the investigated environment through different tissues and organs.

## Materials and methods

### Sample collection

**Fig. 1:**
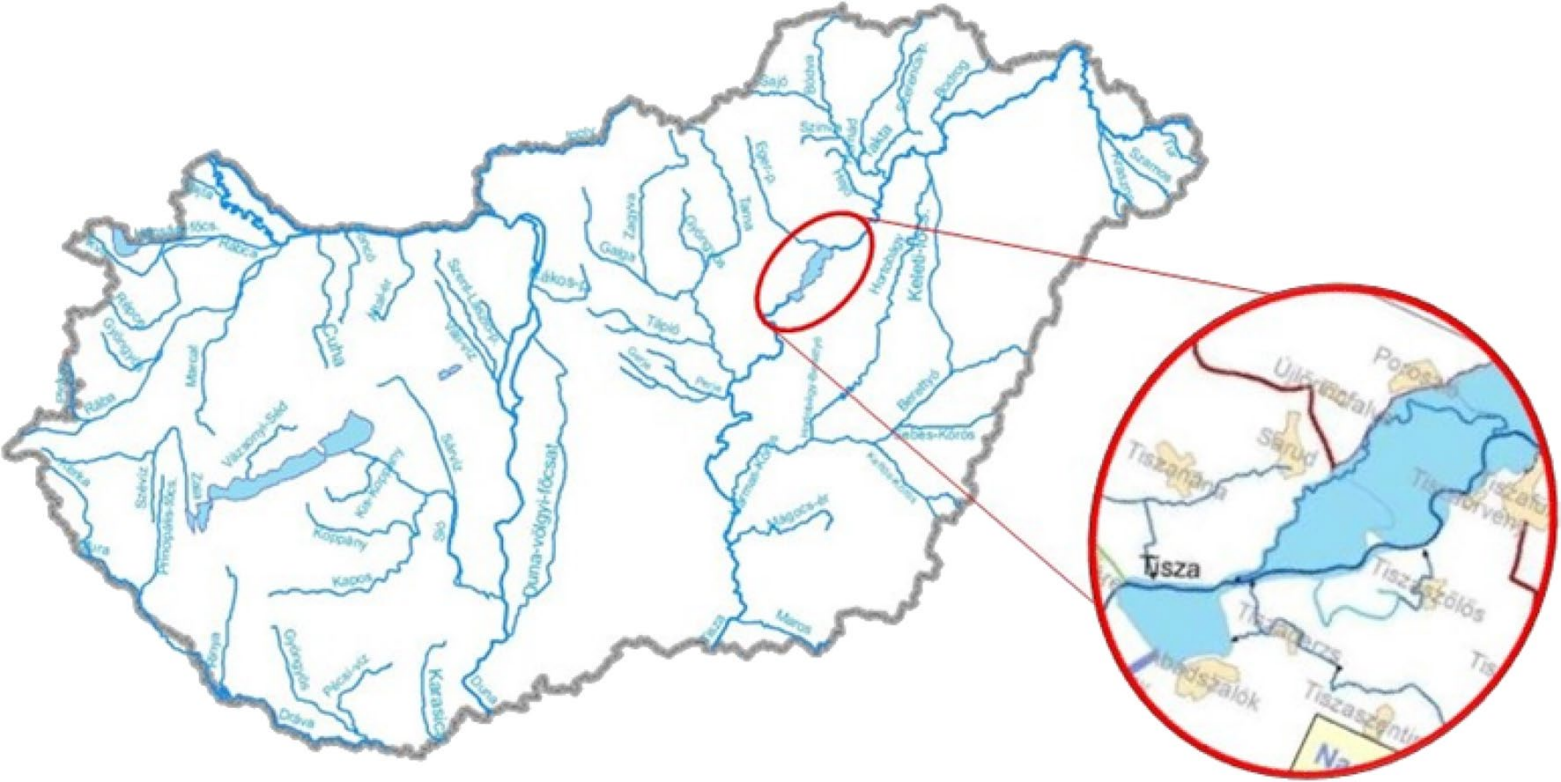
Sampling area

Based on the official permission of the nature conservation permit of the county government office (No. JN-07/61/00253-4/2020), 20 cormorants were shot in the Central Tisza area (on the river section below the dam near Kisköre) in January of 2020 (Decree No. 13/2001 2001), under the supervision of the Nature Conservation Service, due to population management activity to reduce the numbers of cormorants in the region (Fig. 1). Cormorants are non-protected species, which can cause economic losses in the fishery industry.

During the pathological investigation of the age and gender of animals, fish species found in the stomach were investigated and analysed by specialist.

Samples from 20 cormorants of both genders (10 males, 10 females) were taken from feather, liver, kidneys and bones. All samples of liver and kidney (20 g in all cases) and the upper third part of the femur were loaded into individually labelled plastic bags and then all organs of each bird were placed in a common bag. After that, all samples were transported to the laboratory sites in cooler circumstances. They were frozen at −20 °C until analysis. In the case of feathers a mixed sample of primaries, secondaries, and coverts from breast an amount of 5 g was collected by plucking and placed into individually labelled paper bags, and they were stored in a dry, well-ventilated place till the analysis. In all cases a representative sample was taken from them to collect the quantity required for analytical measurement (0.5 g).

### Method

#### Laboratory processing and measurements

The potentially toxic element concentration of the samples was determined in the analytical laboratory of the Department of Animal Hygiene, University of Veterinary Medicine using a Perkin Elmer Optima 3300 DV inductively coupled plasma optical emission spectrometry (ICP-OES) as described by Grúz et al. (2018). During the analysis, the following measurement parameters were applied: RF generator: 40 MHz, RF power: 1500 W, nebulizer type: concentric (Meinhard Type A), nebulizer gas flow rate: 0.9 dm^3^/min, cooling water flow rate: 1 dm^3^/min, sheath gas flow rate: 0.9 dm^3^/min, sample feeding flow rate: 0.9 cm^3^/min, and observation height: 15 mm. The detected wavelengths of each element are shown in Table 1.

**Table 1:** Results of validation

Element	Wavelength of detection (nm)	Calibration curve parameters			Limit of quantitation (ppm)	Limit of detection (ppm)	Precision (%)	Trueness (%)
		Equation (*y* = *a*·*x* + *b*) (1)		(2)				
		*a*	*b*	*r*				
Arsenic	197.197	1287	0	0.999828	1.67	0.50	12.7	13.6
Cadmium	228.802	63,870	0	0.999529	0.17	0.05	8.4	−10.9
Mercury	253.652	10,030	0	1.000000	1.67	0.50	12.3	8.1
Lead	220.353	6520	0	0.999813	0.67	0.20	3.5	−8.4

(1) “*y*” means the signal of the target element at the given concentration level; “*x*” means the concentration

(2) Regression coefficient

#### Analytical standards used in sample processing

Calibration was performed with ICP multi- and mono-element standards (Perkin Elmer Inc., USA; VWR International Ltd., England). The measurements were performed with argon gas of 4.6 purity (Messer Hungarogáz Kft). Quality control (QC) standards were prepared from standard bovine liver reference material NIST SRM 1577c (National Institute of Standards and Technology, Gaithersburg, MD, USA).

### Sample preparation

The feathers were washed in deionised water and ethanol (50 v/v%), to remove adherent exogenous contamination before the analytical procedure. The bones were cleaned from muscle and tendon, and they were broken to sample the required amount for the analytical procedure. After the homogenisation of the tissues, 0.5 g of each sample was weighted into a CEM MARS6 MARSXPreSS teflon vessel for sample digestion. Then they were decomposed by 5 mL nitric acid (69 m/m%) and 5 mL hydrogen peroxide (30 m/m%) in a microwave digestion system (ramp: 35 min; temperature: 200 °C; hold: 50 min; *E*: 1700 W). The sample was filled up with ultrapure water to 25 mL and analysed by inductively coupled plasma optical emission spectrometry (ICP-OES) after a double dilution of deionised water using 1 mg/L Y solution as internal standard and 0.25 mg/L Au for the stabilisation of Hg content.

Blank and the quality control (QC) samples were prepared by the same method. Internal quality control of the measurements was carried out via measuring QC samples of known heavy metal concentration at least 10 times (NIST 1577C-standard bovine liver). After discarding the extremes, the standard deviation of data (SD) was established, which must have remained within the ±15% of the nominal concentration value in order to accept the QC measurement. Every sample, calibration and blank solutions were analysed by 3 replicates.

### Validation of the analytical method

For assessing the reliability of the analytical method and sample preparation, several validation parameters were established according to the relevant guidelines (Commission Decision 2002). Limits of detection (LOD or decision limit, CCα) and limits of quantitation (LOQ or detection capability, CCβ) were defined as three and 10 times the standard deviation of the signals of the blank samples, respectively. Precision was determined as the relative standard deviation of the signals from 10 replicates of the same sample. Trueness was determined by analysing certified reference material (standard bovine liver NIST SRM 1577c) then adding the solution of the four target elements with known concentration (50 μg/kg each) to the same certified reference material as well as comparing and evaluating the analysis results. Both precision and trueness were expressed in percentages. Precision values were accepted below 20%; trueness was accepted in the deviation of the measured parameter that did not exceed ±15%. Linearity was evaluated by the equations of the calibration curves. Matrix effect was not studied since the yttrium solution used as internal standard provided compensation.

The certified Cd content of the reference sample was above the LOD of the method; thus, it was measured directly. The standard deviation and the recovery values are presented (Table 2). The certified values of As, Hg, and Pb were below the relevant LODs; therefore, these parameters were checked by spiking the QC samples to contain additional 0.05 mg/L from the elements (this value is equal to 5.0 ppm calculated in the original sample). The same internal standard was used every time. As the recoveries of all measured elements were within the acceptable range, we classified our sample preparation method overall acceptable. However, we checked the measurement reliability of these elements from another point of view as well. The “percentage of the spiked QC sample” was calculated by dividing the measured spiked sample results by the theoretical results (certified value + 5.0 ppm) and multiply by 100. Spiked QC samples were subjected to the same sample preparation process as all the other samples. In our opinion, this percentage can be used to demonstrate the trueness of method for these elements if we consider trueness as set in the 2002/657/EC Commission Decision.

**Table 2:** Results of quality control (QC) measurement (ppm)

Element	Certified value	Measured value	Spiked QC samples	LOD	Measured/calculated value	Recovery (%)
Arsenic	0.019	n.d.	76.098 ± 0.018	0.500	0.0197	103.5
Cadmium	0.097	0.095 ± 0.006	-	0.050	0.0953	98.3
Mercury	0.005	n.d.	75.875 ± 0.020	0.500	0.0053	106.7
Lead	0.063	n.d.	79.576 ± 0.020	0.200	0.0637	101.1

*n.d.* = not detectable

During the analysis the following potentially toxic elements were analysed in all samples like arsenic (As), cadmium (Cd), mercury (Hg), and lead (Pb). Their limit of detection (LOD) was 0.05 ppm for Cd, 0.2 ppm for Pb, and 0.5 ppm for As and Hg.

### Statistical methods

Statistical analyses were performed using R statistical software (R Core Team 2021).

Samples in which the concentration was below the LOD were calculated as LOD values. The concentrations of As in liver, kidneys, and bones, and that of Hg and Pb in bones, were below the LOD in all samples; therefore, they were not analysed statistically.

Distribution of concentrations was examined with boxplot charts. Independent sample *t*-test was used to compare sexes if normality assumptions were met; otherwise, the nonparametric Mann-Whitney test was applied. Bonferroni correction was used to adjust for multiple tests.

Since statistical difference was not found between the sexes, observations from different sexes were pooled for further analyses. Different tissues were compared using repeated measures ANOVA if its assumptions were met, and the Friedman test was employed otherwise. Normality was checked using boxplots, whilst sphericity using Mauchly’s test. If only normality was met, and sphericity was violated Greenhouse–Geisser correction was applied. The *p*-values were Bonferroni corrected for multiple tests. In the case of significant difference, paired sample *t*-test or nonparametric Wilcoxon test was done with Bonferroni correction for pairwise comparisons.

## Results

### Evaluation of metal concentrations

The concentrations of elements in various types of feathers are different; however, the potential metal contamination of the whole body has been evaluated in this study using different types of feathers taken from several parts of the body, and together with the possible contamination of the environment.

Concentration of As was below detection limit in all samples taken from bones, kidneys, and liver. It was typically above detection limit only in feather samples. Concentration of Cd was above detection limit in all samples except 5% of liver samples. Concentration of Hg was below detection limit in all bone samples and in all other samples as well. Concentration of Pb was below detection limit in all bone samples, 40% of kidney samples, and 45% liver samples.

Mean and 95% confidence interval (95% CI) values can be found in Table 3, where the sexes are pooled.

**Table 3:** Average and 95% confidence interval (95% CI) of metal concentrations measured in liver, kidney, bones, and feathers (unit: ppm)

	Liver				Kidney				Bone				Feathers			
	As	Cd	Hg	Pb	As	Cd	Hg	Pb	As	Cd	Hg	Pb	As	Cd	Hg	Pb
*n*	20				20				20				20			
Average	<0.5	0.129	3.428	0.530	<0.5	0.351	2.926	0.850	<0.5	0.237	<0.5	<0.2	1.222	0.096	4.793	1.721
95% CI	-	0.032	0.705	0.289	-	0.124	0.725	0.449	-	0.021	-	-	0.168	0.020	0.941	0.539
LOD (ppm)	0.5	0.05	0.5	0.2	0.5	0.05	0.5	0.2	0.5	0.05	0.5	0.2	0.5	0.05	0.5	0.2
Ratio below LOD (%)	100	5	0	45	100	0	0	40	100	0	100	100	0	0	0	0

*n* = number of samples

### Comparison of sexes

Since the distribution of the As concentrations was either highly skewed or constant for bone, kidney, and liver samples, so was that of Hg for bone sample and that of Pb for bone, kidney, and liver samples, the comparison was carried out using Mann-Whitney test in these cases. In other cases, where the distributions were close to normal, the comparison was carried out by using *t*-test. There was no significant difference between the sexes at either metal-tissue pair. Therefore, data from different sexes were pooled for further analysis.

### Comparison of the tissue samples

**Fig. 2:**
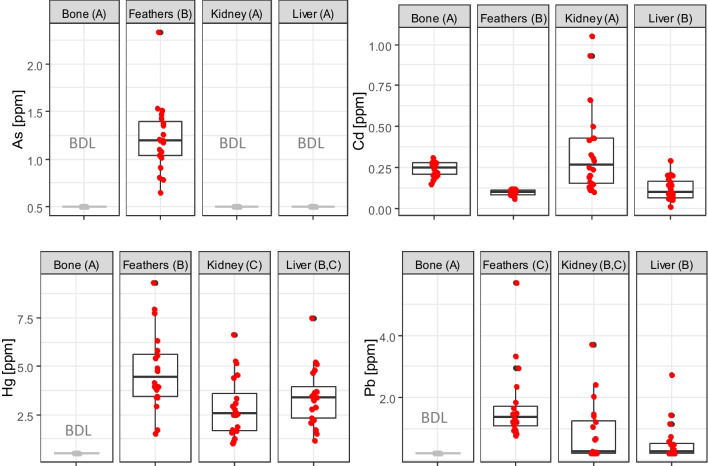
Concentrations for each element-tissue combination. Observations of two sexes are pooled. Individual observations are superposed on boxplots with red symbols. Subplots containing only values below detection limits have been greyed out and denoted with BDL (all samples below detection limit). Letters in parenthesis present the results of pairwise comparisons. Tissues having a common letter do not differ significantly from each other. Tissues having only different letters are significantly different

The comparison of the tissues for As and Pb was carried out by using Friedman test since the distributions were highly skewed or constant (Fig. 2). In case of Hg the constant bone samples were omitted from the omnibus test. Mauchly’s test indicated that the assumption of sphericity was violated for both Cd (*W* = 0.021, *p* = 2e−13) and Hg (*W* = 0.138, *p* = 2e−8), so Greenhouse–Geisser correction was applied to the degrees of freedom to get valid *p*-values.

Difference of the concentration amongst the different tissues was found to be significant for each element. It suggests that every element accumulates in various concentrations in several organs and tissues. Results of the pairwise comparisons can be seen in Fig. 2.

## Discussion

### Arsenic

It is not generally accepted as essential element, but there is evidence that As has an essential and beneficial function. Its elemental form and organic compounds are not toxic, and the acute toxicity of its inorganic compounds is also greatly influenced by chemical structure (Thomas et al. 2001; Laczay 2013; Hu et al. 2020), although in minimal amounts (0.012–0.025 mg/day) it is essential for the body (Sugár and Tóth 2010). Also, Nielsen (1998) suggests that for chicks a determined amount of As is required (12–50-μg/kg diet). Due to the antibiotic and anticoccidial properties of some organic arsenicals, they were used as growth promoters in poultry (Anderson 1983). Czarnecki and Baker (1985) determined that 100-ppm diet as sodium arsenite generates copper accumulation in the kidneys of chicks (100-mg roxarsone/kg diet).

When excessive amounts are ingested, the highest amount of arsenic can be measured in liver and kidneys. Due to the affinity of arsenite to bind sulfhydryl groups in the body (e.g. keratin), high amounts can be accumulated in skin, hair, and nails. García-Cegarra and Martínez-López (2023) measured 0.87 ± 0.12 ppm As in the feather samples of red-legged cormorants (*Phalacrocorax gaimardi*) in Chile. In a study of Einoder et al. (2018), 1.2 ± 0.8 ppm As was detected in black-faced cormorant (*Phalacrocorax fuscescens*) feathers. There are few similar studies using wetland birds’ feather to measure the amount of As, which makes it difficult to determine whether the concentrations presented in this study may have caused any effects. In our tissue samples arsenic was only detectable in the feather samples of the cormorants, but only at very low amount (1.222 ppm), its level was below LOD (0.50 ppm) in liver, kidneys, and bones.

### Cadmium

Cd is non-essential and accumulates in the food net. It can induce damages of different tissue and behavioural problems (Burger and Gochfeld 2000b; Butt et al. 2018). Cadmium after absorption and distribution can be incorporated into zinc-containing proteins resulting in severe damages (Tang et al. 2014).

In birds, 3 ppm is the accepted threshold for Cd in the liver (Scheuhammer 1987; Nighat et al. 2013). However, its concentration close or above to 3 ppm testifies to heavy contamination of the environment. Based on findings derived from other investigations in birds of prey, the adverse effects are as follows: reduced growth rate has been observed in the range of 0.1 to 2 ppm (Spahn and Sherry 1999; Burger 1993; Naccari et al. 2009). Cd is not transferred to eggs efficiently, so its level in egg is much lower than those found in the diet of hens. For example, the Cd level was undetectable in the albumen and 0.1 ppm in egg yolks collected from hens with 100 ppm Cd in liver (Leach Jr et al. 1979; Sato et al. 1997).

When Cd accumulates in bones, it causes different osteodiseases. Even a low level of exposure may promote skeletal demineralisation, which leads to bone fragility and risk of fractures (Bhattacharyya et al. 1988; Silver and Nudds 1995; Scheuhammer 1996; D’Haese et al. 2017; Järup 2002; McFarland et al. 2002). Because Cd has about a 20-year biological half-life, different pathological conditions have been diagnosed in avian species. During the time of exposure, the tissue levels reach a plateau, which occurs rapidly in muscles (within months), but in liver and kidneys for a much longer period. When the exposure ceases, the reduction of Cd from tissues is not too significant, even if the muscles and bones do not accumulate cadmium at high levels (White and Finley 1978; Sharma et al. 1979; Baxter et al. 1982).

Studies in Japan show similarity to our measured data in the liver, where cormorants from the vicinity of Tokyo accumulated 0.28 ppm Cd, and from the region of Lake Biwa 1.25 ppm (Saeki et al. 2000).

In the feathers of red-legged cormorants García-Cegarra and Martínez-López (2023) detected average concentration of Cd as 1.49 ± 0.36 ppm. In black-faced cormorant feathers, this concentration was 1.7 ± 1.2 ppm (Einoder et al. 2018). Mirsanjari et al. (2014) measured 0.02 ± 0.02 ppm in feathers of great cormorant in Iran. Compared to our results there is a higher concentration of Cd in kidney (18.56 ± 2.46 ppm) and in the liver (4.13 ± 0.59 ppm) in yellow-legged gulls (*Larus michahellis*) (Vizuete et al. 2022).

In our study the highest mean Cd level (0.351 ppm) was detected in the kidneys of cormorants, followed by the bones (0.237 ppm), liver (0.129 ppm), and feather (0.096 ppm), which correlates to the findings of other studies that Cd accumulation is the greatest in kidneys amongst the organs (NRC 2005). Cd concentrations in kidneys and bones were significantly higher than in liver and feather, whilst the bones vs kidneys and feather vs liver concentrations were not significantly different from each other.

### Lead

Lead is a non-essential element and has various adverse effects on the CNS, renal, hematopoietic, neurologic, cardiovascular, and gastrointestinal systems (Charkiewicz and Backstrand 2020). It can cause liver, skin, and lung cancer; changes haematological parameters; can also cause cerebral oedema, neuronal damage, demyelination, anaemia, and bone marrow suppression; and decreased peripheral nerve conduction peripherally. Significant suppression of growth can be caused by 1 ppm Pb in the diet (Bakalli et al. 1995). Also, behavioural changes occur (such as screaming), because the bird is in pain or otherwise uncomfortable (Bakalli et al. 1995). By experience it has been found that 4 ppm Pb in feather can induce delayed parental, locomotor, and feeding behaviour of seagulls (Burger 1995) and more adverse effects of lead can occur above 4 ppm (Burger and Gochfeld 2000c) whilst 2 ppm Pb in liver and 10–20 ppm (d.w.) in bones results in subclinical poisoning (Pain et al. 1995, 2005).

Lead is retained by soft tissues and eventually by bone and the excretion is very slow through the kidneys (Rădulescu and Lundgren 2019). In the body of the animals Pb levels in bones are the highest, followed by kidneys and liver. Lower Pb concentration is detected in skeletal muscle.

In great cormorants’ feathers the average concentration of lead was 0.67 ± 0.24 ppm in the study of Mirsanjari et al. (2014). Higher concentration was detected by García-Cegarra and Martínez-López (2023) 2.82 ± 0.96 ppm in red-legged cormorants’ feathers, and by Irena et al. (2017) in the feather samples of great cormorants (2.18 ± 0.74 ppm), and an even higher one in black-faced cormorant feathers, 10.6 ± 5.9 ppm (Einoder et al. 2018). In liver samples of great cormorants collected at a nature conservation area of Kis-Balaton, the Pb concentration was 0.670 ± 0.221 ppm (Lehel et al. 2013), which is similar to our results.

Compared to our data, Agusa et al. (2005) detected lower lead concentration in the liver and the kidneys of black-tailed gulls (0.05 ppm and 0.25 ppm).

Similar findings to ours have been observed in a study from Spain by Vizuete et al. (2022), where they measured higher concentration of Pb in kidneys (2.50 ± 0.78 ppm) than in liver (0.55 ± 0.77 ppm) in samples from yellow-legged gulls; 4.38 ± 1.09 ppm in the feathers of yellow-legged gulls was detected, and it is also a higher level compared to the 0.83 ± 0.37 ppm in the same species (Otero et al. 2018), or the 0.399 ± 0.048 ppm in Northern gannet (*Morus bassanus*) (Nardiello et al. 2019). Vizuete et al. (2019) and Nardiello et al. (2019) stated that Pb values are higher in the kidney compared to liver and feathers (in yellow-legged gulls and in northern gannet), which is contradictory to our findings.

Our results as opposed to the abovementioned literature data showed the highest mean concentration in the feather (1.721 ppm), followed by kidneys (0.850 ppm), liver (0.530 ppm), and in bones the concentration in all samples was below the detection limit. The feather vs liver, feather, and kidney concentrations were significantly different. But all the measured Pb levels in these tissues are below the abovementioned thresholds.

### Mercury

Mercury is a non-essential heavy metal, one of the most toxic and persistent heavy metals in the aquatic ecosystem (Nguyen et al. 2005). In the environment, some mercury is transformed to methylmercury, which is more toxic, by bacteria and fungi during various biological processes (Wood and Wang 1983). This reaction occurs primarily in aquatic systems (Gworek et al. 2020). Thus, methylmercury of microbial origin is able to enter the food chain and accumulate in animals; it is highly toxic and persistent, which can cause problems in the food web (Bloom 1992; Nguyen et al. 2005; Rodríguez-Estival et al. 2020). During long-term administration, young chickens tolerated 1.35 ppm of mercury without growth problems (March et al. 1983), but 5 ppm had already increased their mortality; in ducks 3.8 ppm caused behavioural changes (Soares Jr et al. 1973; Bhatnagar et al. 1982).

The majority of mercury load (70–93%) in the body of birds can be accumulated in feathers (Braune and Gaskin 1987; Burger and Gochfeld 1997; Bond and Diamond 2009), because of the keratin content of the feathers, as methylmercury has a high affinity for sulfhydryl groups. According to the literature, Hg binds to the matrix of the feathers, cannot be mobilised and dissolved from it, so the only route of mercury excretion is moulting (Goede and de Bruin 1984; Burger 1993; Dauwe et al. 2002).

Burger and Gochfeld (2001) observed very low level of Hg (0.251 ppm) in the feather of Cape Cormorant (*Phalacrocorax capensis*) from Namibia. García-Cegarra and Martínez-López (2023) detected a similar low concentration in the feathers of red-legged cormorants (0.66 ± 0.04 ppm). Misztal-Szkudlińska et al. (2010) analysed great cormorants’ feathers, where the total Hg concentrations in contour feathers were 9.73 ± 5.63 ppm, whilst in tail feathers were 6.43 ± 4.21 ppm. Furtado et al. studied imperial cormorant (*Leucocarbo atriceps*) and their feather samples showed 2.69 ± 0.77 ppm concentration of Hg. Higher concentration was detected in great cormorants (13.14 ± 4.89 ppm) by Irena et al. (2017) and in black-faced cormorants (19.3 ± 6.9 ppm) by Einoder et al. (2018).

In yellow-legged gulls the Hg concentrations of kidney (2.94 ± 0.18 ppm) and liver (2.95 ± 0.21 ppm) levels of Hg were similar, but in the feathers the highest metal concentration was Pb (4.38 ± 1.09 ppm), not Hg (1.13 ± 0.08 ppm), compared to the present study (Vizuete et al. 2022).

In a study on great cormorant tissue samples show higher concentrations in kidney (3.79 ± 0.71 ppm) and in liver (5.71 ± 1.85 ppm) (Aazami 2018). Similarly, to our data, in the liver and kidney samples of great cormorants 3.4 ± 1.1 ppm and 2.5 ± 2.2 ppm Hg were measured by Nam et al. (2005). Skoric et al. (2012) studied the bones of great cormorants and measured 1.04 ± 0.22 ppm of Hg in adult birds.

In a previous study on great cormorants from Hungary at a nature conservation area of Kis-Balaton, Hg concentration in liver samples of adult birds was 4.479 ± 3.336 ppm (Lehel et al. 2013).

In the present study, mostly lower concentration was detected than in the above-mentioned sources. The highest mean Hg levels were in the feathers (4.793 ppm), followed by liver (3.428 ppm) and kidneys (2.926 ppm), and in bones were below LOD. The concentration in feathers is significantly higher than in the bones, liver, and kidneys.

In our study the detected concentrations match well with the MTL (Maximum Tolerable Level) data of these elements (Table 4) (NRC 2005). Our results show that the investigated area is not contaminated with potentially toxic elements to such an extent that could lead to chronic exposure or could even adversely affect the growth, reproduction, or behaviour of cormorants.

**Table 4:** Comparison of potentially toxic elements in feather, liver, kidney, and bone tissue samples (average, ppm) and MTL values (ppm feed)

Element	Liver	Kidney	Bone	Feather	MTL value
Arsenic	<0.50	<0.50	<0.50	1.222	30
Cadmium	0.129	0.351	0.237	0.096	10
Lead	0.530	0.850	<0.20	1.721	10
Mercury	3.428	2.926	<0.50	4.793	5.0

## Conclusion

The burden of potentially toxic elements in the organs and tissues of birds is mostly influenced by various factors, as the accessibility of these elements, the quality of their food, and the metal burden in their nesting and feeding area. Based on our results the measured quantities of the examined elements stored in the liver, kidneys, bones, and feather samples of great cormorants do not exceed levels indicative of poisoning, even if the most exposed birds are the ones at the top of the food net, such as cormorants, and show that the tested area is not contaminated at a level that can cause adverse effects or poisoning in birds. In addition, it points out that these elements are presented in the environment and should be regularly monitored to be able to detect and analyse their increase of these to avoid possible pollution/poisoning in the future.

### Supplementary information

ESM 1 (DOCX 14 kb)

## Data Availability

The datasets used and/or analysed during the current study and the supplementary materials are available from the corresponding author on reasonable request.
