# Supplementary material for: Potentially toxic elements in different tissues of great cormorant (Phalacrocorax carbo) at a wetland area
Source: Environ Sci Pollut Res Int. 2023 Nov 9;30(57):120540–51. doi: 10.1007/s11356-023-30791-3 (PMC10697887; doi:10.1007/s11356-023-30791-3)
Supplement: Supplementary file 1 — (DOCX 14 kb) [file 11356_2023_30791_MOESM1_ESM.docx]

Supplementary file prepared based on the request of JEO Assistant Carmina Joy Cayago

Our previously submitted manuscript (ESPR-D-22-16161) was rejected by the Editor. The letter below was received from the Editor-in-Chief

"Dear Dr. Budai,

Thank you for submitting your manuscript.

Unfortunately, and after a careful examination of your paper, I am sorry to inform you that your manuscript is not acceptable for a publication in ESPR in the present format.

Therefore, I must reject the manuscript as is, but I encourage you to resubmit as a new manuscript after the appropriate changes or research have been completed.

My reason in consideration for your paper is that the bibliography part must be updated only few cited references in the last 5 years.

Yours sincerely,

Prof. Dr. Philippe Garrigues

Editor-in-Chief

Environmental Science and Pollution Research"

**Our response**: The bibliographic part of our new manuscript (ESPR-D-23-02464) has been updated with more references from the last 5 years based on the proposal of the Editor-in-Chief.
